# Facial paint contamination: A new removal technique by using paraffin oil

**DOI:** 10.1097/MD.0000000000043394

**Published:** 2025-07-11

**Authors:** Xiao-Xue Tan, Dan-Dan Hou, Nan Xu, Jie-Feng Huang, Min Xu, Ya-Hong Zhu

**Affiliations:** a Department of Nursing, The First Affiliated Hospital of Zhejiang Chinese Medical University (Zhejiang Provincial Hospital of Chinese Medicine), Hangzhou, Zhejiang, China; b Department of Orthopaedics & Traumatology, The First Affiliated Hospital of Zhejiang Chinese Medical University (Zhejiang Provincial Hospital of Chinese Medicine), Hangzhou, Zhejiang, China; c Department of Emergency Center, The First Affiliated Hospital of Zhejiang Chinese Medical University (Zhejiang Provincial Hospital of Chinese Medicine), Hangzhou, Zhejiang, China.

**Keywords:** facial paint contamination, paint removal, paraffin oil

## Abstract

Facial paint contamination is a common issue, and traditional removal methods, such as turpentine, can be irritating, particularly in sensitive areas such as the eyes and mucous membranes. There is a pressing need for safer and more effective alternatives for paint removal from the facial skin. This retrospective study aimed to evaluate the effectiveness of paraffin oil in removing facial paint stains. A total of 6 patients with facial paint contamination were treated at the emergency center of The First Affiliated Hospital of Zhejiang Chinese Medical University between March 2021 and October 2023. Paraffin oil was applied to the contaminated areas using sterile cotton balls or gauze, and gentle rubbing was performed until the paint was removed. Patients with concomitant skin lacerations received appropriate treatment, while those with paint in sensitive areas (e.g., eyes, nose) were referred to relevant specialists. Paraffin oil proved to be an effective agent for paint removal, with no allergic reactions or complications observed. The average time required for paint removal ranged from 27.5 to 30 s/cm². The procedure was well-tolerated, and no adverse effects were noted. Using paraffin oil provided a safe, rapid, and nonirritating method for removing facial paint, with minimal sensitization risk.

## 1. Introduction

With rapid economic growth, paint usage has become increasingly widespread. A survey in a major city revealed a higher rate of health abnormalities among paint-industry workers, associated with occupational exposure.^[1,2]^ In clinical practice, removing paint contaminants from the skin remains an important and challenging problem. In some cases, paint contamination occurs on the face, delicate mucosal areas, or within skin lacerations, which increases the difficulty of treatment.

At present, methods for paint removal include laser ablation,^[3,4]^ electro-oxidation,^[5]^ and the use of ionic liquids.^[6]^ However, there is a lack of practical guidelines and robust evidence for efficiently decontaminating skin. Existing techniques all have shortcomings: facial skin and mucous membranes are easily damaged, and the nose, mouth, and eyes are particularly sensitive to irritating odors.^[7]^ Therefore, we explored the use of paraffin oil to remove paint, aiming to overcome these deficiencies.

## 2. Materials and methods

### 2.1. Study design

This study was approved by the hospital’s ethics committee, and all participants provided informed consent. We recruited patients treated with paraffin oil in the emergency center of our hospital between March 2021 and October 2023.

Inclusion criteria were: facial paint contamination; age ≥18 years; and provision of informed consent.

Exclusion criteria were: cognitive impairment or psychiatric disorder; treatment with any non-paraffin oil paint-removal method; history of dermatologic allergy (e.g., urticaria); and severe or multiple injuries requiring cardiopulmonary resuscitation or endotracheal intubation. Detailed patient characteristics are summarized in Table 1.

**Table 1:** General information of participants (n = 6).

Item	Age (yr)	Gender	Injury time (h)	Paint contaminated	Area of paint adhesion	Combined with skin laceration	Types of wound contamination	Treating	Outcome	Time consumed (s)	Clearance efficiency (s/cm²)
1	43	Male	0.4	Facial area	10 cm × 7 cm	No	–	Cleaning	No allergy	525	7.50
2	51	Male	2	Facial area	5 cm × 8 cm	Yes	Dirty	Debridement and suturing, oral antibiotics for 3 d	First intention healing	323	8.08
3	49	Male	0.5	Facial area	6 cm × 8 cm	Yes	Dirty	Debridement and suturing, IV antibiotics for 3 d	First intention healing	487	10.15
4	55	Male	1	Facial area	4 cm × 5 cm	No	–	Cleaning	No allergy	218	10.9
5	53	Male	1.5	Facial area	5 cm × 5 cm	No	–	Cleaning	No allergy	299	11.96
6	48	Female	0.6	Facial area	7 cm × 6 cm	No	–	Cleaning	No allergy	403	9.59

### 2.2. Technique

**Figure 1.**
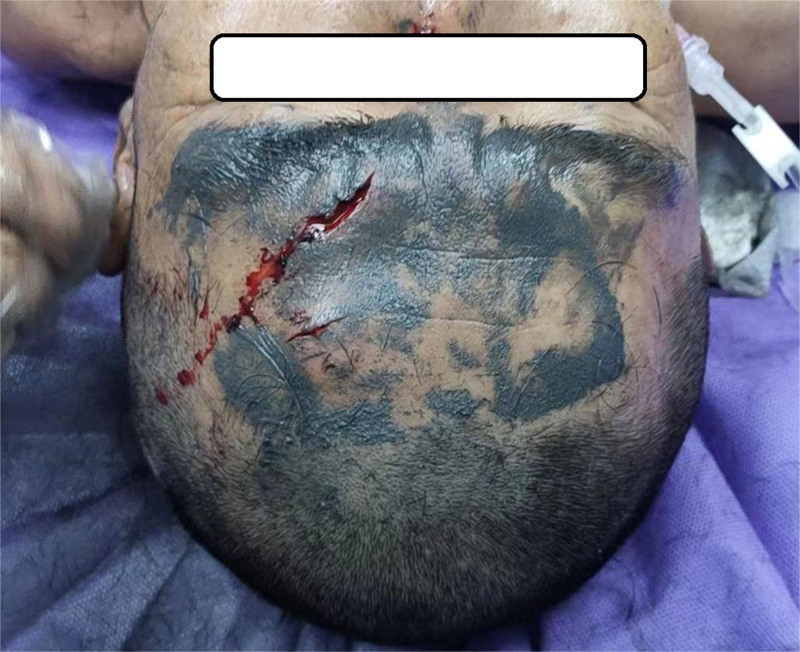
There was a skin laceration about 2 cm in size stained with black paint on the forehead.

**Figure 2.**
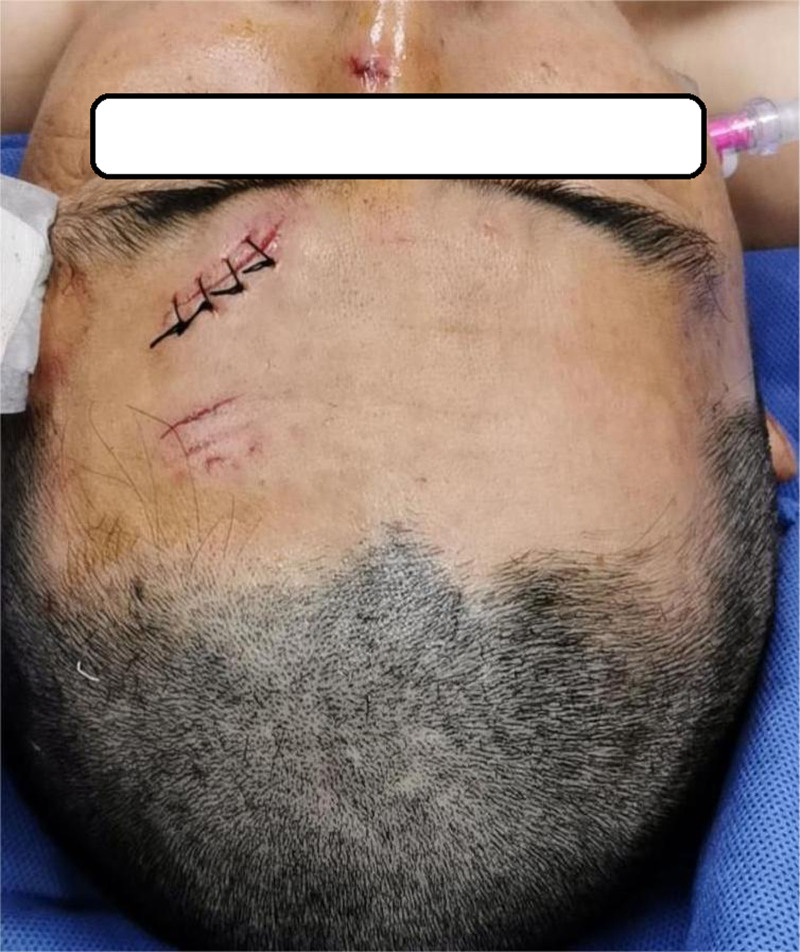
Skin condition after paint removal, saline rinse, debridement and suture.

With the patient in the supine position, sterile gauze or cotton balls soaked in paraffin oil were applied in a Z-shaped pattern, extending beyond the edges of the contaminated area, and then gently wiped until visible paint was removed. The treated area was then irrigated with saline for at least 1 minute. In cases with concurrent skin lacerations (all treated within 6 hours of injury), primary debridement and suturing were performed once the wound appeared clean. Oral or intravenous antibiotics were administered as clinically indicated. Skin was monitored for allergic reactions for 15 to 30 minutes post-application. For contamination of sensitive sites (eyes, nasal cavity, ears, or mouth), relevant specialists were consulted, and paraffin oil application to mucous membranes was performed under their guidance (Figs. 1 and 2).

### 2.3. Data collection

Researchers recorded the site and area of paint contamination, time to clearance, any allergic reactions, and complications. Demographic and clinical data (age, sex, diagnosis) were extracted from electronic medical records. Two investigators independently verified all data and entered them into a 3-column table on the day of collection.

### 2.4. Statistical method

All analyses were conducted using Statistical Package for the Social Sciences (SPSS) 26.0 (IBM Corp., Armonk). Continuous variables conforming to a normal distribution are presented as mean ± standard deviation.

## 3. Results

Six patients with facial paint contamination were included in this study. The mean age was 49.83 ± 4.22 years; 5 were male and 1 was female. Two patients presented with concurrent skin lacerations and underwent debridement and suturing, whereas the remaining 4 underwent cleansing only. Additional patient details are provided in Table 1.

## 4. Disscussion

In clinical practice, turpentine and alcohol are commonly used to remove paint and adhesives. However, turpentine’s unpleasant, pungent odor and its terpene content are major contributors to allergic reactions.^[8]^ Inhalation of turpentine vapors in large quantities may cause headaches, nausea, and tremors. Alcohol can also trigger allergic responses due to Aldehyde dehydrogenase 2 polymorphisms common in East Asian populations.^[9]^ Both turpentine and alcohol are known to induce severe ocular irritation and may provoke contact dermatitis, underscoring the need for caution and protective measures when treating sensitive areas such as the face, eyes, and mucous membranes.

These limitations led us to explore paraffin oil as an alternative. Our findings indicate that paraffin oil is a gentle yet effective solvent: it dissolves and removes paint without irritating mucous membranes or exacerbating skin or wound damage. It can be safely applied to facial areas and, in most cases, promotes uneventful wound healing. Paint-removal efficiency – measured as 9.69 s/cm² on average^[10]^ – suggests that paraffin oil can expedite decontamination, thereby reducing overall treatment time, patient discomfort, and risk of complications.

Paraffin oil is commonly used in skin care to soothe and treat rough skin, acne, dark spots, and blemishes. Regular application of paraffin oil can increase blood flow and collagen production, promoting a more youthful skin appearance. However, the saturated hydrocarbon constituents of paraffin oil are chemically inert and exhibit minimal reactivity toward cutaneous proteins and enzymes, resulting in an exceedingly low incidence of allergic responses. Nonetheless, individuals with oily or acne-prone skin should perform a preliminary patch test on a small area before widespread application.

In our cases, paraffin oil treatment resulted in primary intention healing (cases 2 and 3), suggesting not only effective paint removal but also enhanced wound healing compared with turpentine and alcohol. Irritants such as turpentine and alcohol should be avoided when treating open wounds, as they may cause pain, inflammation, delayed healing, increased infection risk, and hinder recovery. In this study, no patients developed allergic reactions, consistent with previous reports of low allergenic potential for paraffin oil.^[11]^ Paraffin oil consists of nonpolar molecules that solubilize polymeric residues; both paint and adhesive contaminants are polymers. Additionally, literature suggests that fatty acids in oils can compromise the performance and stability of paint films.^[12]^ We found that this mechanism can be translated into clinical practice to address paint contamination on wound sites, facilitating cleaning and suturing while minimizing hazards posed by residual paint.^[13]^ To our knowledge, this is the first clinical report of this method.

Although paraffin oil demonstrated excellent efficacy and safety for removing facial paint contamination, its high viscosity and occlusive properties may limit its suitability for individuals with oily or acne-prone skin. Excessive application in these skin types could contribute to follicular occlusion, increased sebum retention, or acne exacerbation. While no adverse dermatological reactions were observed in this study, the small sample size and limited diversity of skin types warrant caution when generalizing these findings. Future studies should assess the tolerability, long-term safety, and clinical applicability of paraffin oil across various skin types, particularly among populations with seborrheic or acne-prone conditions.

This study have several limitations. The small sample size may introduce potential bias, and the homogeneity of our cohort limits external validity. We did not analyze the chemical composition of the paint, and all data were collected from a single center. Future prospective, multicenter research is necessary to evaluate paraffin oil’s paint-removal efficacy under diverse environmental conditions and in broader patient populations, thereby providing a more robust scientific basis for its clinical application.

## 5. Conclusion

Paraffin oil proved to be an effective, safe, and nonirritating method for removing facial paint contamination, avoiding the toxicity and irritation associated with traditional solvents such as turpentine and alcohol. This study offers a promising clinical alternative for decontamination in sensitive facial areas, providing a gentler treatment with minimal risk of adverse reactions.

## Author contributions

**Conceptualization:** Min Xu, Ya-Hong Zhu.

**Data curation:** Dan-Dan Hou.

**Formal analysis:** Nan Xu.

**Investigation:** Dan-Dan Hou.

**Methodology:** Nan Xu, Jie-Feng Huang.

**Software:** Xiao-Xue Tan.

**Supervision:** Ya-Hong Zhu.

**Writing – original draft:** Xiao-Xue Tan.

## References

[1] Sekhar L Govindarajan Venguidesvarane A Thiruvengadam G. Respiratory symptoms and pulmonary function in paint industry workers exposed to volatile organic compounds: a systematic review and meta-analysis. PLoS One. 2024;19:e0315464. 39724031 10.1371/journal.pone.0315464 PMC11671006

[2] Office of the Secretary HUD. Requirements for notification, evaluation and reduction of lead-based paint hazards in federally owned residential property and housing receiving federal assistance; response to elevated blood lead levels. Fed Regist. 2017;82:4151–72. 28102982

[3] Zhang L Zhou K Feng G. Influence of water environment on paint removal and the selection criteria of laser parameters. Chin Phys B. 2022;30:423–33.

[4] Zhang D Xu J Li Z. Removal mechanism of blue paint on aluminum alloy substrate during surface cleaning using nanosecond pulsed laser. Opt Laser Technol. 2022;149:107882.

[5] Can-Güven E Guvenc SY Ilhan F. Application of combined EO/PMS/Me²⁺ process in organic matter and true color removal from paint manufacturing industry wastewater. Environ Res. 2022;212(Pt C):113451. 35537495 10.1016/j.envres.2022.113451

[6] Sarwono A Man Z Idris A. Alkyd paint removal: ionic liquid vs volatile organic compound (VOC). Prog Org Coat. 2018;122:79–87.

[7] Aldelaimi AA Ahmed RF Enezei HH Aldelaimi TN. Gummy smile esthetic correction with 940 nm diode laser. Int Med J. 2019;26:513–15.

[8] Tanaka H Ikai E Yamada Y. Genetic polymorphisms in alcohol metabolizing enzymes as related to sensitivity to alcohol-induced health effects. Environ Health Prev Med. 1997;1:193–200. 21432474 10.1007/BF02931216 PMC2723532

[9] Li J Wang J Wu M. Observation of the effects of paraffin oil and turpentine on the removal of 3M adhesive tape residue from the skin. Nurs Pract Res. 2008;5:25–6.

[10] Osmond G. Zinc white and the influence of paint composition for stability in oil-based media. Issues Contemp Oil Paint. 2014:263–81.

[11] Karlberg AT Lepoittevin JP. One hundred years of allergic contact dermatitis due to oxidized terpenes: what we can learn from old research on turpentine allergy. Contact Dermatitis. 2021;85:627–36. 34453446 10.1111/cod.13962

[12] Biribicchi C Doutre M Favero G. Assessing the swelling behavior of oil paint in fatty acid methyl esters (FAMEs). RSC Adv. 2024;14:39692–9. 39691226 10.1039/d4ra07464e PMC11650782

[13] Boranbayeva L Boiko G Didukh A. Combined effect of depressor additive and heat treatment on the rheological properties of highly paraffinic oils. ACS Omega. 2025;10:7783–94. 40060853 10.1021/acsomega.4c08126 PMC11886638

